# Triangulating Truth and Reaching Consensus on Population Size, Prevalence, and More: Modeling Study

**DOI:** 10.2196/48738

**Published:** 2024-03-19

**Authors:** Ian E Fellows, Carl Corcoran, Anne F McIntyre

**Affiliations:** 1 Division of Global HIV & TB Global Health Center Centers for Disease Control and Prevention Atlanta, GA United States

**Keywords:** HIV, epidemiology, population size estimation, key populations, Bayesian models, consensus estimation, statistical tool, prevalence, Bayesian model, population, estimate, consensus, population size

## Abstract

**Background:**

Population size, prevalence, and incidence are essential metrics that influence public health programming and policy. However, stakeholders are frequently tasked with setting performance targets, reporting global indicators, and designing policies based on multiple (often incongruous) estimates of these variables, and they often do so in the absence of a formal, transparent framework for reaching a consensus estimate.

**Objective:**

This study aims to describe a model to synthesize multiple study estimates while incorporating stakeholder knowledge, introduce an R Shiny app to implement the model, and demonstrate the model and app using real data.

**Methods:**

In this study, we developed a Bayesian hierarchical model to synthesize multiple study estimates that allow the user to incorporate the quality of each estimate as a *confidence* score. The model was implemented as a user-friendly R Shiny app aimed at practitioners of population size estimation. The underlying Bayesian model was programmed in Stan for efficient sampling and computation.

**Results:**

The app was demonstrated using biobehavioral survey-based population size estimates (and accompanying confidence scores) of female sex workers and men who have sex with men from 3 survey locations in a country in sub-Saharan Africa. The consensus results incorporating confidence scores are compared with the case where they are absent, and the results with confidence scores are shown to perform better according to an app-supplied metric for unaccounted-for variation.

**Conclusions:**

The utility of the triangulator model, including the incorporation of confidence scores, as a user-friendly app is demonstrated using a use case example. Our results offer empirical evidence of the model’s effectiveness in producing an accurate consensus estimate and emphasize the significant impact that the accessible model and app offer for public health. It offers a solution to the long-standing problem of synthesizing multiple estimates, potentially leading to more informed and evidence-based decision-making processes. The Triangulator has broad utility and flexibility to be adapted and used in various other contexts and regions to address similar challenges.

## Introduction

One of the more daunting tasks for policy makers in the public health arena is the synthesis of data and information into actionable insights. Population size, prevalence, and incidence are crucial metrics that play a significant role in shaping public health programs and policies. However, stakeholders are often faced with multiple estimates of these quantities, originating from different sources and of differing quality. Often, estimates of the same quantity will be incompatible, with confidence bounds completely disjoint from one another.

Stakeholders face the task of weighing their knowledge about the population along with the various estimates to triangulate to a single number that represents the most likely value. Typically, this has been done without a formal framework, where decisions about how the final number was arrived are shrouded by a nontransparent process. How much stock was each estimate given by the stakeholders? Did any of the stakeholders express strong prior beliefs about what the true number was that could affect the result? Did political considerations color the findings, and if so, how? Without transparency, these questions are difficult to answer.

The goal of this study is to present a statistical tool that can be used to guide the triangulation process. Prior beliefs, study quality, and estimate uncertainty are all components of the process of generating a consensus estimate. However, because these components are put together in a rigorous statistical framework, all of them are made completely transparent and inspectable by third parties.

A particular focus is paid to population size estimation (PSE). PSE is one of the fundamental estimates required for policy decisions in HIV treatment and prevention. Understanding the population size is essential for determining the appropriate scale of the public health response. Without this knowledge, it becomes challenging to tailor interventions effectively. This is especially true for key populations (KP), which are less visible groups disproportionately at risk for HIV, such as female sex workers (FSW), men who have sex with men (MSM), and people who inject drugs [1]. Globally, KP and their sexual partners account for more than half of the new HIV infections as of 2019 [2]. Reliable estimates of KP sizes are needed to inform the planning and implementation of prevention and treatment programs as well as to assess the outcomes of these control measures. In particular, evaluating progress toward the Joint United Nations Programme on HIV/AIDS 95-95-95 goals (95% of people living with HIV know their status, 95% of those who know their status are receiving antiretroviral therapy [ART], and 95% of those receiving ART are virally suppressed) [3] requires accurate population size estimates.

Although these estimates have significant implications, there is currently no gold standard method for PSE [4]. In the absence of such a standard, various techniques have been developed, each with varying degrees of rigor [5]. On the one hand, estimates can be based on nonempirical data such as the opinion or experience of subject matter experts, such as the wisdom of crowds [6,7] or the Delphi method [8,9]. On the other hand, empirical data–driven biobehavioral survey (BBS)–based techniques including service or unique object multipliers [10] and recruitment information captured in surveys using respondent-driven sampling [11] can provide more reliable population size estimates [12] but can still produce incongruous results [13]. Moreover, CIs only capture part of the uncertainty we have in an estimate. Mistakes in implementation, violation of method assumptions, and incorrect statistical model application can all add additional bias and uncertainty that are not captured by the CI. A major question is how to synthesize multiple estimates of varying quality and certainty, given the practical need of policy makers and scientists for a single *best estimate* of population size. Although a range of estimates with uncertainty bounds derived from multiple statistical methods may be a more comprehensive snapshot, multiple estimates are often challenging to interpret if they are disparate and difficult to apply to problem-solving or performance evaluation. To address this issue, a consensus *best estimate* is often arrived at somewhat arbitrarily by round table discussions of subject area experts; however, the absence of empirical evidence in such processes makes them susceptible to bias or statistical errors.

Recently, more rigorous statistical methods have emerged for finding a consensus-based estimate based on approaches in the field of meta-analysis. Meta-analysis is a branch of statistics concerned with synthesizing the results of multiple studies that aim to estimate the same quantity, often an effect size, in medical or epidemiological studies [14]. Traditional meta-analysis approaches have been based in frequentist statistics, although Bayesian techniques are becoming more common. Table 1 shows a component of the spectrum of frequentist models in meta-analysis for combining the effects of studies. In the fixed effects model, it is assumed that each study estimates the same effect, and only within-study variation is incorporated. In the random effects model, each study is assumed to estimate a different effect drawn from a population distribution and thus incorporates between-study variation. However, both models take the results of each study at face value. With the goal of incorporating bias adjustment into more traditional models, the *quality effects model* [15] has recently been introduced, and it has been shown to have fewer limitations than competing models [16]. In the quality effects model, each study considered is appraised on its methodological quality and given a quality score, which is then used to adjust the study variance within the model. Although other meta-analysis methods have Bayesian PSE analogs, such as the *Anchored Multiplier*, which draws on both fixed and random effects models [17,18], the quality effects model has yet to be adapted for use triangulating public health quantities.

In this study, we present a Bayesian hierarchical model to synthesize multiple study estimates that allow the user to incorporate the quality of each estimate as a study *confidence* score. We developed a mathematical framework for the model as well as a metric to assess the variation between estimates that have been captured by the model. We also introduced an implementation of the model as a user-friendly R Shiny app aimed at practitioners of PSE. To demonstrate both the model and the app, we provide several examples of their use in combining KP size estimates in several cities in a country in sub-Saharan Africa, showing the potential of our model to provide a valid *best estimate* in a practical context.

**Table 1:** Meta-analysis pooled effects modelsa.

	Fixed effects	Random effects	Quality effects
Weights	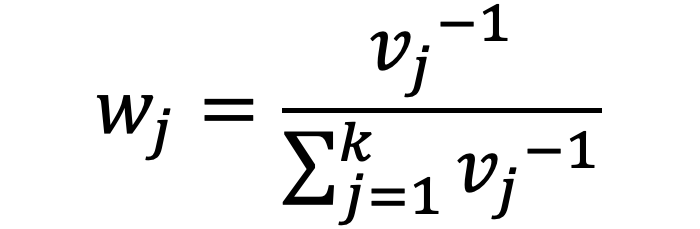	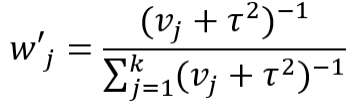	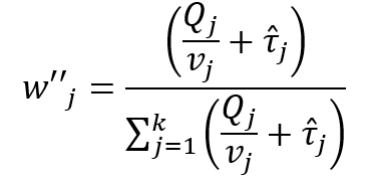
Estimator	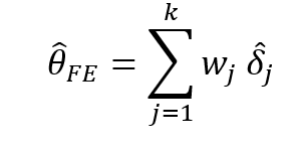	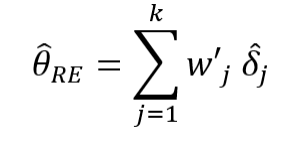	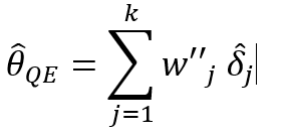
Estimator variance	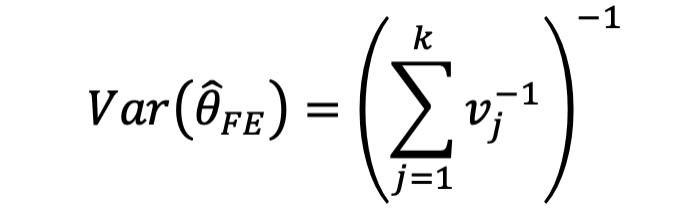	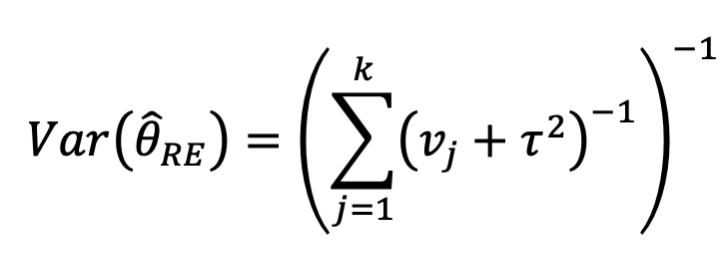	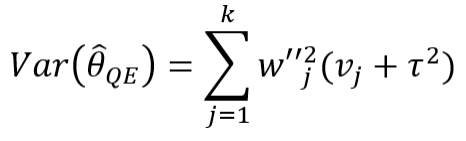

^a^
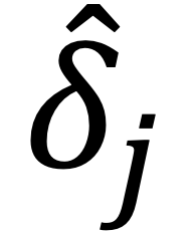
 is the estimated effect size of *j*th study, *v_j_* is the sampling error variance of *j*th study, τ^2^ is the DerSimonian and Laird estimator of the between-study variance, *Q_j_* is the *jth* study rank (scaled between 0 and 1), and 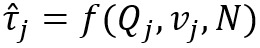 is a bias correction (where *N* is the number of studies).

## Methods

### Overview

We began the construction of our model by considering a Bayesian hierarchical model that is fundamental in meta-analysis [19]. The primary goal of such a meta-analysis is to estimate the mean of the distribution of effect sizes, synthesizing the estimates of individual studies. Our goal was similar: to estimate a population quantity of interest θ which can be a population size, population proportion, incidence, among others. We aimed to synthesize several estimates *y_j_* of this quantity from different studies, where the variance *σ_j_*^2^ of each estimate is known.

Despite the assumption that the variance *σ_j_*^2^ is known (ie, has been computed during the processing of the *j*th study), it may not truly reflect the uncertainty in the estimate of *y_j_*. The variance *σ_j_*^2^ only accounts for the sampling uncertainty and not any additional uncertainty that there may be about potential bias in the study design. For example, in PSE, certain size estimation techniques result in incongruous estimates and nonoverlapping CIs [13], indicating that there is additional nonsampling error not accounted for by the CIs and *σ_j_*^2^. Nonsampling bias can be introduced for a number of reasons, including study design assumption violation, a mismatch between the study population and the population of interest, or an out-of-date sample. Our model provides a simple way for the user to account for their assessment of the degree of this additional uncertainty in the study. We allow the model user to adjust this uncertainty based on their confidence level *c_j_*∈(0,1) in the study, where *c_j_*=1 represents full confidence in the study’s estimate and uncertainty. The SD is scaled by the transformation *σ_j_*→*σ_j_*/*c_j_* to adjust for this additional uncertainty at the discretion of the user. This allows practitioners and experts, whose expertise may carry information about each estimate not accounted for in the given CIs, to manually incorporate the potential for study bias.

The synthesis itself is based on a Bayesian hierarchical model that is often used in meta-analysis. At the first level of the hierarchy, we assume that each *y_j_* is a point estimate of the quantity *v_j_* estimated by the *j*th study:

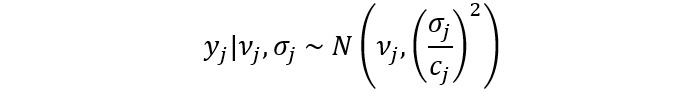



A normal model is chosen because many point estimates tend to be asymptotically normal according to the central limit theorem. That the *y_j_* are estimates of *v_j_*, and not θ directly, is an important distinction; individual studies may have bias, and that bias may not be accounted for by the user-specified study confidences. At the second level of the hierarchy, we assume that each study’s quantity of interest *v_j_* itself is centered around the true population quantity of interest θ with uncertainty characterized by the between-study variance τ^2^. At this level, we assume that the *v_j_* are distributed normally as

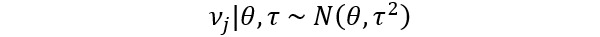



Although the *v_j_* provide a bridge between the data *y_j_* and the target quantity θ, the between-study variance τ^2^ provides an important source of uncertainty beyond the adjusted study-level variances (*σ_j_*/*c_j_*)^2^. When τ^2^ is small, the *v_j_* are similar, and most of the uncertainty in the model comes from the data level; when τ^2^ is large, most of the uncertainty comes from differing *v_j_*. However, from a practical perspective, the *v_j_* represent a nuisance parameter that can significantly slow down the computational model. Eliminating this parameter by marginalizing it out yields the *reduced model* (refer to Multimedia Appendix 1 [18] for details):

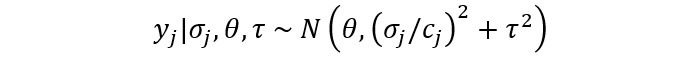



In collapsing the hierarchical model to a single level, we can quickly and efficiently infer the mean of the distribution of the quantity of interest, given our data θ|*y_j_*.

### Prior Distribution for θ and τ

As this is a Bayesian model, we must consider the distribution of the hyperparameters θ and τ^2^. In general, we assume a normal prior for θ with mean *μ*_0_ and variance *σ*_0_^2^:

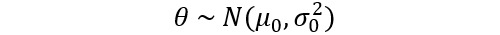



The specification of this prior distribution for θ is an important part of the inference process, as experts and stakeholders will often have informed beliefs about reasonable values for the population quantity.

Setting a prior distribution for τ^2^ is more complex, as there are multiple candidate distributions, each with advantages and disadvantages; refer to the study by Williams et al [20] for discussion of the choice of prior on τ and why it may be desirable to place the prior on τ instead of τ^2^. We choose a half-Cauchy distribution for our prior on τ, which is a Cauchy distribution truncated so that only positive values have a nonzero probability density. The distribution is heavy tailed, which is important because the model must allow for the possibility of large between-study variance. That said, we wish to trust that our stakeholders have accounted for the additional study variability in the confidence score step and thus wish to construct an informative prior that puts most of its mass at a very small τ^2^ but which allows for it to be large if the data are incompatible with a small τ^2^. This has the effect of flexibly controlling the model complexity, with the *v_j_* values only being appreciably different from θ if the data require it.

We control this distribution with a single-scale parameter *γ*, and thus, we choose that parameter to accumulate most of the probability mass for τ^2^ at 0. We use an empirical Bayes–flavored choice, which is scaled to be a fraction of the sample SD of the estimates *y_j_*:

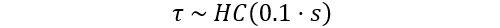



where *s*^2^=Var(*y_j_*). As a heuristic justification for the choice of 0.1 as the value of the multiplier on the sample SD, we note that the quantile function for a half-Cauchy distribution with scale parameter *γ* is 
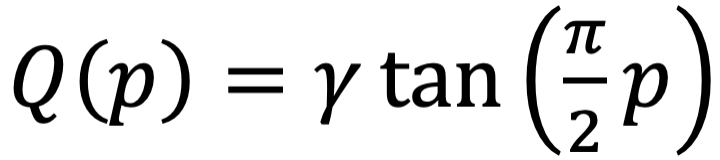
, and thus, the 95th percentile is given by Q(0.95)≈12.7*γ*. As such, with *γ*=0.1*s*, a little more than 95% of the probability mass of τ is below the sample SD *s* which we have found is often sufficient to create a high burden for values of τ^2^ away from 0.

### Transforming the Data and Parameter Space

The hierarchical model assumed a normal distribution for the estimates. This is generally justifiable, as most estimates follow the central limit theorem and are therefore approximately normal if the sample size of the study is large enough. However, depending on the quantity, the central limit theorem may be applied to smaller sample sizes when transformed. Regardless, the Delta method provides some assurance that transformed point estimates are asymptotically normal if the untransformed estimate is also asymptotically normal.

For instance, a log transform is typically used in PSE, where log-linear models are a prime example. Models of proportions often involve a logistic transformation, as seen in techniques such as logistic regression. With our model, the user may apply a transformation to the data, run the model, and then apply an inverse transformation to obtain inferences regarding the quantity of interest.

Working in a transformed space also transforms the prior, which can be desirable in and of itself. The log transform of PSE causes the prior to be log-normal. This may be a more reasonable prior distribution for this quantity because it is always >0. Similarly, for the logistic transform, the prior becomes a logistic normal distribution whose values are bounded from 0 to 1.

Transformations may induce infinities in some edge cases (eg, a point estimate of zero or one with a logistic transformation). In these cases, either the estimate may be dropped, or the analyst can choose not to use a transformation.

### Relationship With Frequentist Meta-Analysis Models

The frequentist methods in Table 1 describe increasingly complex models for estimating the combination. The fixed effect model simply calculates a weighted average of the estimates, weighted by the inverse variance of each estimate. Our model is the Bayesian analog of this fixed effect model when all study confidences are set to 100% and τ→0. For τ>0 and the study confidences set to 100%, our model is the Bayesian analog of the frequentist random effects model, where the τ in the random effects model is approximately analogous to our τ. By positing a heavy-tailed prior for τ highly concentrated at 0, our model leans toward a fixed effect model when the data are compatible with it and a random effects model when the data are not compatible with it.

The quality effects model introduces a researcher-defined quality metric to the random effects model in much the same way that our *study confidence* does. Thus, when study confidence was included, the features of the quality effects model were incorporated.

### Explained Variance and Unaccounted-For Variation

Although we use the reduced model for computational reasons, the full multilevel model contains information about the sources of uncertainty that may have a significant impact on the confidence a user has in the resulting estimate of θ. To quantify the sources of uncertainty, we computed a metric called *explained variance*, or *R*^2^, which describes each level of the full model [21]. In the full multilevel model, *R*^2^ calculated at the study (data) level is defined as

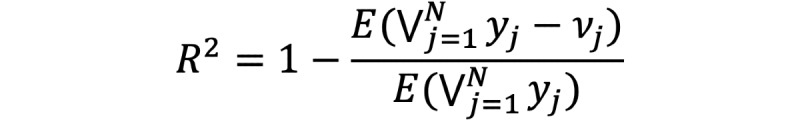



where E(⋅) the posterior mean and 
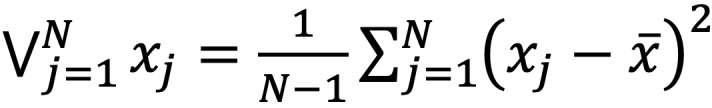
 is the sample variance operator. From this definition, an observation can be made: when the *v_j_* more closely approximate the *y_j_*, *R*^2^ is closer to 1, and when the *v_j_* more closely approximate θ, *R*^2^ is closer to 0. In the case where there is considerable variation in the estimates *y_j_*, *R^2^* reflects the proportion of estimate variability attributable to unaccounted-for study bias. When the variation in the estimates is largely owing to unaccounted-for study bias, the *v_j_* will approximate the *y_j_*, leading to high *R*^2^. When study bias is accounted for by increasing the uncertainty of the estimates, the *v_j_* will more closely approximate θ, leading to low *R*^2^. To reflect this relationship, we use the term *unaccounted-for variation* to refer to *R*^2^.

A complication with the abovementioned formula is that it relies on the parameters *v_j_* that have been eliminated in the reduced model. However, we can compute *R*^2^ at the study level using the data and posterior draws of θ and τ^2^ that are specific to the model at hand. We find that

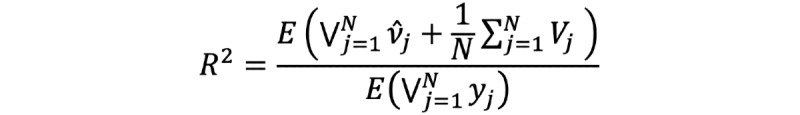



where 
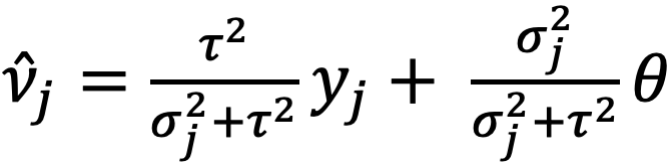
 and 
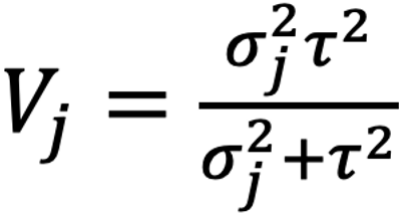
.

A full derivation of the preceding formulas can be found in Multimedia Appendix 1 [18].

### Example Using BBS-Based PSE Methods

#### Overview

The population size estimates used to demonstrate the Bayesian hierarchical model using the Shiny app were all part of a 2019 BBS conducted among FSW in 3 cities (location A, location B, and location C) and MSM in 2 cities (location B and location C) of a country in sub-Saharan Africa. Priors and corresponding plausibility bounds were based on nonempirical stakeholder consensus during the 2014 BBS. In each survey city and for each key population (FSW and MSM), 2 methods of PSE were implemented: multipliers and successive sampling.

#### Multipliers

As part of a BBS, unique object, event, and service multipliers provide data sources to facilitate PSE. Multipliers have 2 overlapping components, both of which include members of a target population such as FSW or MSM. For each of the 3 multipliers, the first component takes place shortly before the BBS implementation. For unique object multipliers, small, inexpensive, and memorable unique objects (*gifts*) are distributed to target populations by teams of key population peers within the survey catchment area. For event multipliers, a party or concert is hosted for target population members. For service multipliers, client lists from HIV service providers are cleaned and deduplicated. The counts are tallied of individuals who received a gift, attended an event such as a survey kick-off party or concert, or accessed HIV services from a particular provider during defined dates. The second component is the weighted proportion of BBS respondents who reported receiving a gift, attending an event, or accessing HIV services. A population size estimate results when the counts are divided by the weighted proportion. These are reported with the corresponding 95% CIs.

For FSW in all 3 locations and MSM in location C, the survey multipliers included a unique object distributed in the survey catchment areas, a special event for the target population, 2 providers of testing services, and 1 outreach provider. The MSM survey at location B included an additional fourth outreach service multiplier.

#### Successive Sampling

As part of a BBS using respondent-driven sampling for recruitment, participants were asked a series of questions to determine their personal network size that informs the survey weighting. One assumption is that individuals with larger social networks are more likely to be sampled and more socially visible and, thus, recruited into surveys earlier than those with smaller networks [22]. The successive sampling PSE (SS-PSE) method has been described elsewhere [23] and applies this assumption to self-reported personal network size and the order of recruitment to estimate the population size. The SS-PSE used in this study uses imputed visibility to adjust for errors in network size reporting [24]. The SS-PSE was generated for FSW and MSM in all survey locations.

#### Confidence Scores

The purpose of the confidence score is to quantify, on a scale of 0 to 100, the quality or reliability of each individual PSE generated by various methods. It aims to provide a measure of how much trust or confidence one should place in each PSE. Confidence scoring requires knowledge of the methods and quality of PSE. Elements to consider for each PSE include whether the definitions of the populations are aligned; when (ie, Were the various studies conducted concurrently as part of the same BBS? Within 1 to 2 years of each other? Several years apart?) and how each of the PSE methods were implemented and analyzed; and how to apply that information to each PSE independent of, not relative to, other PSE. We have more confidence in empirical methods implemented concurrently in the same catchment area among populations with the same or similar characteristics, such as age range, sex, residence, and behaviors, with high-quality, complete data and minimal errors. Therefore, we assigned a high score that reflects the confidence we have in the PSE derived from an activity meeting these criteria. In contrast, if we have data from similar but not the same populations, conducted several years apart and sampling different catchment areas, with some missing data, we have less confidence in those PSE and assign a low score. Adjusting confidence scores or dropping low-quality PSE to produce a specific, desired outcome is strongly discouraged.

All PSE methods are subject to errors during implementation. Unique object, service, and event multipliers are commonly included with BBS. The first of the multipliers (or, if sampled twice before a survey, 3-source capture-recapture) involves offering unique objects to key population members in hot spots, typically carried out by teams of their peers (eg, FSW teams distributing unique objects to fellow FSW in venues where they congregate, often referred to as *hot spots*). The selection of which hot spots to visit (geographic coverage), who is responsible for distributing the unique objects (ideally, peers within the key population to increase acceptability), and the distribution method used (eg, hasty, clustered distribution vs a more systematic approach to peers in the hot spot) can vary significantly, potentially introducing bias into the data collection process. Broad geographic coverage implemented by peers using a systematic or random approach to distribute enough (eg, twice the survey sample size) unique objects would earn a high score. Next, health service client lists may be outdated (ie, include former or expired clients), contain duplicates, or exclusively cover a portion of the population (eg, an ART client registry representing only individuals who are HIV positive and actively receiving treatment). These challenges would result in a low score. Events may draw only a specific subgroup of the target population or fail to attract enough participants, which may compromise the reliability of the PSE. This was particularly apparent when the PSE produced by the event multiplier was smaller than the survey sample size. This scenario would result in a nonplausible PSE and a very low score.

In respondent-driven sampling surveys, participants were tasked with providing information regarding their personal network size. This can present a significant challenge for respondents to estimate, as it is a function of the clarity of survey questions and a respondent’s ability to quantify the size of their network quickly and accurately. The response may not reflect the actual size. Even after adjusting the self-reported network size for social visibility, the resulting data could potentially impact the subsequent PSE during the successive sampling process. Confidence scores were independently applied to each PSE method and reflected the quality of implementation based on the elements described in this section as well as the plausibility of the estimates.

### Ethical Considerations

The BBS survey was reviewed and approved by the ministry of health in the sub-Saharan African country; the implementing partner; and the Centers for Disease Control and Prevention Global Health Center in Atlanta, United States. The data collection staff completed training on human subjects research and signed a confidentiality agreement before commencing their survey duties. All participants provided written informed consent.

## Results

### Confidence Scores

We reviewed all PSE methods for FSW and MSM at each survey location and assigned a confidence score (Table 2). The confidence score was based on our knowledge of the rigor of the methods used for PSE and the quality of implementation of those methods. The scores were assigned to each method independent of other methods implemented in the survey; thus, a confidence score for one method was neither relative nor comparative to another method. Confidence scores were selected from the interval (0,100), with 100 representing full confidence. These scores were then divided by 100 to yield the confidence score *c_j_*∈(0,1), as described in the *Methods* section.

**Table 2:** Survey-based population size estimates by location and key population.

		Confidence score	Population size estimate (95% CI)				
			FSW^a^ in location A	FSW in location B	MSM^b^ in location B	FSW in location C	MSM in location C
Prior		—^c^	800 (300-2000)	3000 (1800-3400)	2416 (850-4000)	900 (825-1500)	610 (475-685)
**Estimate method**							
	Unique object	80	679 (525-1024)	382 (228-949)	221 (174-904)	690 (483-1191)	379 (280-681)
	Event	5	162 (126-249)	144 (69-354)	96 (81-205)	205 (146-378)	122 (87-276)
	Service 1	60	849 (630-1369)	1984 (1033-7367)	515 (384-4398)	1540 (1181-2260)	2322 (1158-6985)
	Service 2	60	2766 (1995-4622)	4459 (3082-10,219)	1917 (3493-1218)	2937 (2127-4613)	1917 (1394-4093)
	Service 3	60	668 (500-1082)	5449 (1769-30,997)	778 (660-5256)	2004 (1138-5916)	156 (97-494)
	Service 4	60	N/A^d^	5900 (2674-45,597)	N/A	N/A	N/A
	SS-PSE^e,f^	70	674 (318-2426)	2196 (1651-2382)	2205 (382-10,409)	1057 (576-3369)	664 (405-1614)
Consensus: confidence scaled^e^		—	890 (614-1342)	2744 (2046-3768)	1412 (727-3111)	1038 (781-1333)	612 (517-730)
Consensus: unscaled^e^		—	729 (403-1335)	2821 (2080-3856)	1638 (812-3416)	922 (701-1205)	606 (508-724)

^a^FSW: female sex workers.

^b^MSM: men who have sex with men.

^c^Not applicable (this refers to the fact that confidence scores are only applicable to the estimates).

^d^N/A: not applicable (this refers to the fact that an estimate of type *Service 4* was not collected).

^e^95% CI refers to the credible interval.

^f^SS-PSE: successive sampling population size estimation.

The implementation of PSE methods was similar across all survey locations for FSW and MSM, so our confidence scores were applied consistently by method. This was appropriate because our FSW and MSM populations had the same population definitions across survey sites and the methods were concurrently and consistently implemented. Had any survey site or population struggled with implementation, that site and the PSE method would have been assigned a lower score. At all survey sites, enough unique objects were broadly distributed across the survey catchment area, and the PSE from unique object distribution in FSW and MSM hot spots was plausible; thus, we assigned a high confidence score of 80. Providers of HIV testing and outreach services reviewed and used similar methods to clean their client lists; however, the services did not represent the entire FSW or MSM population in any of the cities, only those who pursued testing or engaged in outreach services. The service providers were unable to guarantee that the lists contained only current clients (ie, no former or expired clients) and were fully deduplicated; thus, they were assigned confidence scores of 60. The service providers varied by key population and city but were provided with the same instructions on how to clean and deduplicate the client lists, so the confidence scores were applied consistently for FSW and MSM services across all cities. Finally, all event multipliers indicated low overall participation, with only a small proportion of survey respondents reporting attendance. The events were similar in content and attendance for FSW and MSM across the survey cities. The resulting PSE for FSW and MSM in each survey city was smaller than the survey sample sizes. Despite being a low-quality, nonsensical PSE, the results were not dropped from the model; they were assigned a low confidence score of 5. The distributions of self-reported personal network sizes for FSW and MSM in each survey city were plausible with a few outliers; therefore, we adjusted for social visibility to reduce the impact of those outliers on the PSE. The SS-PSE using imputed visibility for FSW and MSM was consistent with previous study results and program data, so SS-PSE was assigned a high confidence score of 70.

### Using the Shiny App

The confidence-scaled consensus estimation model is deployed in an easy-to-use Shiny app that requires only a few inputs from the user. There are 3 pages on the app: *Enter Estimates*, *Define Prior Beliefs*, and *Synthesis*.

As an example, we consider estimating the size of the population of FSW in location A of our example country in sub-Saharan Africa. The 6 PSEs and the prior are all specified in Table 2. The results of the model after running the app are also included in Table 2.

The prior estimate for the population is 800 FSW, with plausible bounds of (300, 2000). This prior was arrived at by consensus among stakeholders and experts based on the 2014 BBS-based population size estimates using nonempirical methods such as the wisdom of crowds and literature reviews. The relatively wide bounds cover most of the new PSEs, which are all similar except for the *Event* and *Service 2.* The low *Event* estimate is mitigated by its low confidence, and as such, it will have less of an effect during synthesis. However, the high *Service 2* estimate has relatively high confidence, so we would expect this estimate to pull the posterior mean higher.

**Figure 1:**
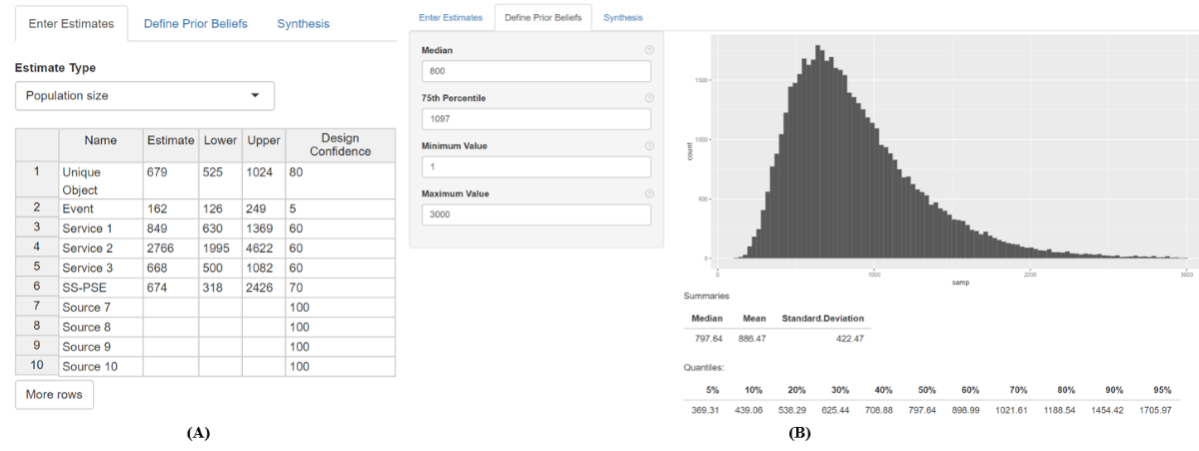
The (A) Enter Estimate tab and the (B) Define Prior Beliefs tab of the Shiny app. The Enter Estimates tab requires the user to input any number of estimates, upper and lower bounds of the 95% CIs of those estimates, and confidence scores (out of 100) as well as input the estimate type to determine the most appropriate transformation of the input data. The Define Prior Beliefs tab requires users to input the median and 75th percentile for the prior distribution of the quantity being estimated and optionally maximum and minimum values that the quantity estimated can take on.

The estimates are entered in the *Enter Estimates* tab of the app (Figure 1A) along with the upper and lower bounds of the CI and the confidence in the study (ranked out of 100). The other choice that the user must make on this tab is whether to apply a transformation to the data, with the options being no transformation, log transformation, and logit transformation. The log transformation is recommended when working with population size estimates, whereas the logit transformation is recommended for percentage or proportion data.

The prior beliefs are then entered in the *Define Prior Beliefs* tab (Figure 1B). The median of the prior should match the prior estimate of the population size. The 75th percentile, which is used to determine the spread of the prior, is also required. This is meant to be a practical and flexible way to characterize the uncertainty in the prior. If the user has reliable information about the prior uncertainty, such as the 95% CI, the 75th percentile can be computed and entered. If the user does not have reliable information about the uncertainty, they may try several options for the 75th percentile, and the app will resample to create the prior distribution shown in Figure 1B until the user believes the distribution shown reasonably reflects their knowledge of the population size.

The lower and upper bounds on the prior are not required for the model to run but can speed up the computation and provide a more reliable synthesis of the estimates by ruling out impossible prior values. In this example, the lower bound is set to 1 because there must be at least 1 FSW to make the population size worth estimating. The upper bound is set to 3000, which is somewhat arbitrary but makes improbably large population sizes unlikely.

Once prior information has been entered, the model can be run on the *Synthesis* tab. One additional input is the τ *multiplier,* which helps to set the scale parameter for the prior on τ. We have found that this default works well in most cases, although it is possible that it may be appropriate to change it in some circumstances. For instance, one might wish to decrease the multiplier (eg, to 0.01 or 0.001) if there is good reason to believe that all PSEs are of precisely the same population, such as if the studies were done at the exact same time with the exact same catchment area. In this case, barring errors in the implementation of the studies, the between-study variance would be expected to be close to 0, and thus, the prior on τ can be adjusted to make high values of τ unlikely. In contrast, if less is known about the implementation of the studies and there is reason to believe that there might be some true between-study variance, the multiplier could be increased (eg, to 1) to allow τ to take on larger values or to make the prior less informative.

**Figure 2:**
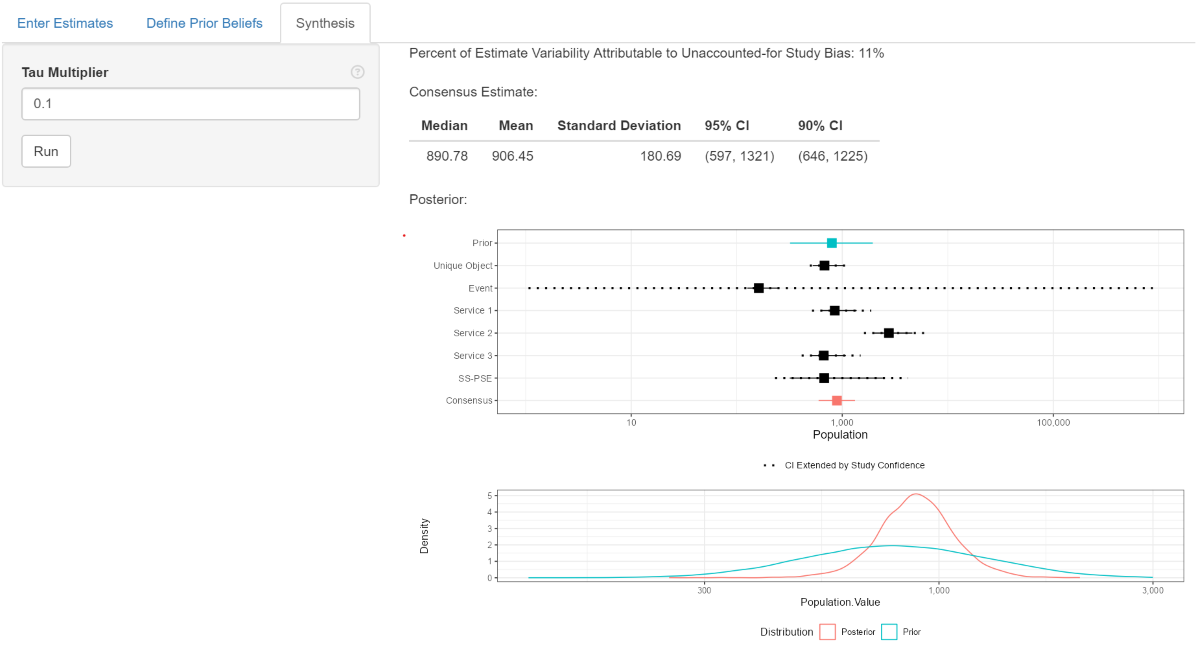
The Synthesis tab of the Shiny app, which includes the consensus estimate and a forest plot of the prior, the estimates, and the consensus estimate as well as the unaccounted-for variation. For the estimates in the forest plot (black), the solid lines represent the unscaled 95% CI, whereas the dotted lines represent the confidence-scaled 95% CI.

The results show both the prior and the posterior distributions of the population size θ as well as descriptive statistics for the posterior distribution of θ (Figure 2). Notably, the posterior median was higher and the uncertainty was lower when compared with the prior. The observation that the posterior variance is smaller than the prior variance is generally true: even if the PSEs are given very low confidence (and thus very high uncertainty), the posterior will look very similar to the prior. Refer to the *Discussion* section for further observations on this point. This tab also reports the unaccounted-for variation at the study level (*R*^2^) as the “percent of estimate variability attributable to unaccounted-for study bias.” In this example, *R*^2^=11%, indicating that the confidence scaling in the model accounts for most of the nonsampling error that explains the differences among the study estimates. This is compared with the unscaled case (Table 2), where *R*^2^=92%, indicating the incongruity of the estimates. A forest plot of the prior, estimates, and consensus results (for both confidence scaled and unscaled) is also shown in Figure 2, with both the reported CIs for the estimates (solid) as well as the scaled CIs (dotted).

### Other Cases

**Figure 3:**
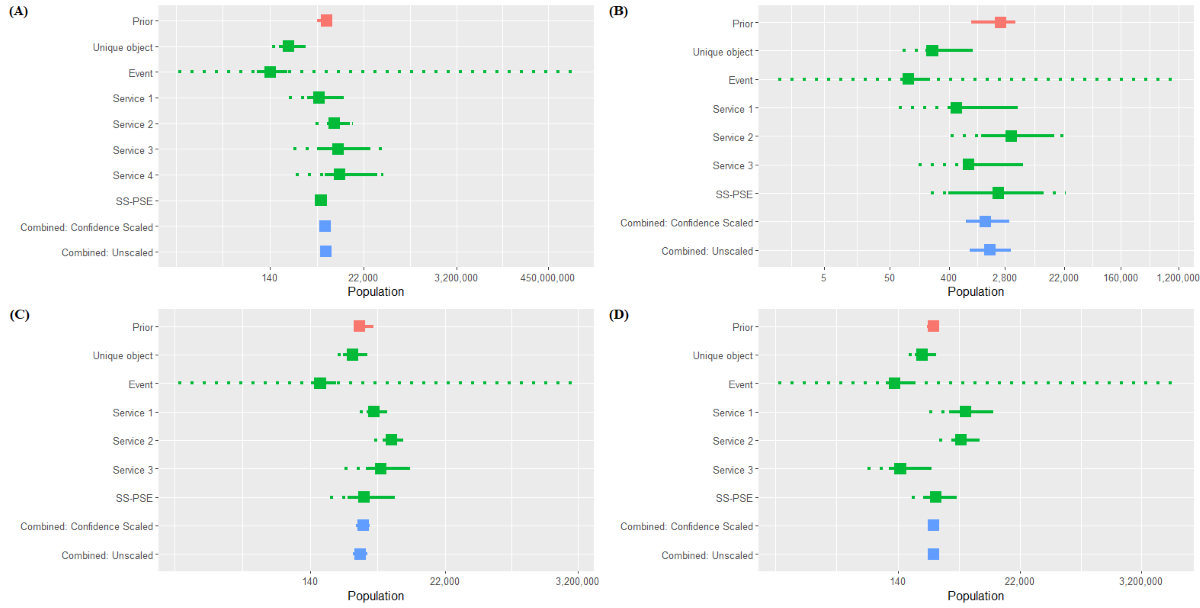
Forest plots of prior distribution of the true population size θ, population size estimates, and consensus estimates for (A) female sex workers (FSW) in location B, (B) men who have sex with men (MSM) in location B, (C) FSW in location C, and (D) MSM in location C. Consensus estimates include unscaled confidence (ie, all study confidence levels set at 100) and confidence scaling (with confidence scores given in Table 2).

**Figure 4:**
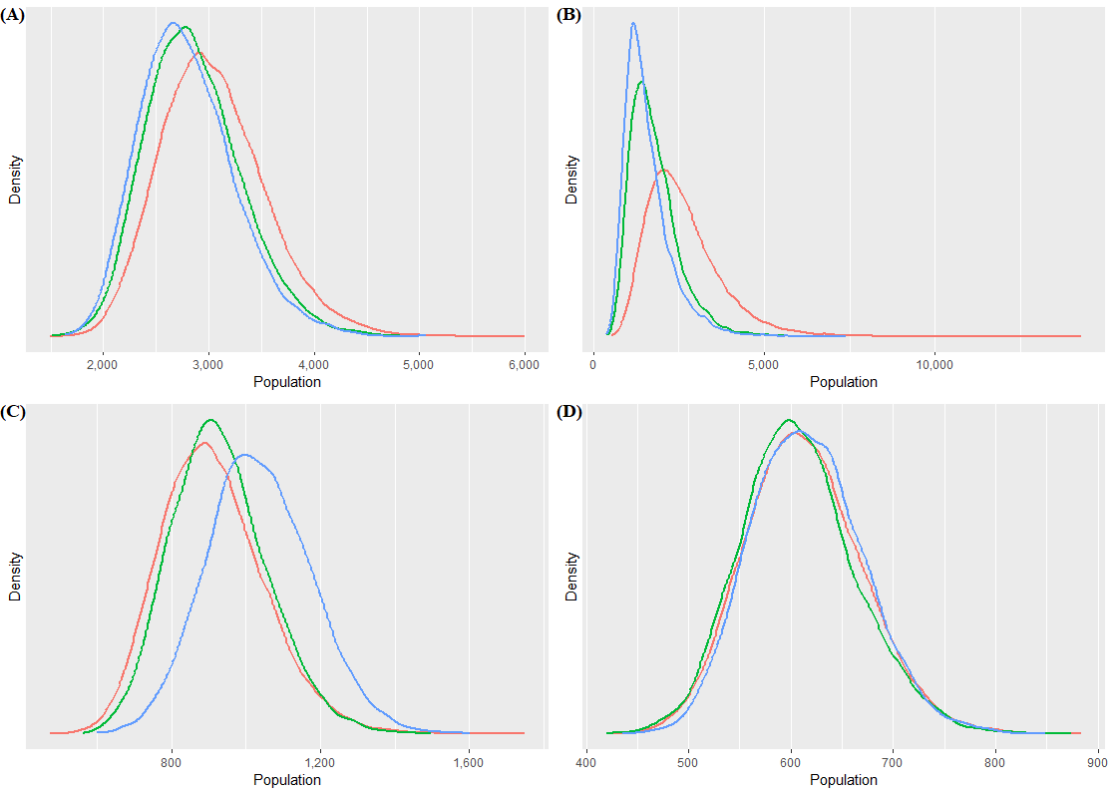
Prior and posterior distributions of the true population size θ for (A) female sex workers (FSW) in location B, (B) men who have sex with men (MSM) in location B, (C) FSW in location C, and (D) MSM in location C. The prior is shown in red, the posterior with unscaled confidence (ie, all study confidence levels set at 100) is shown in green, and the posterior with confidence scaling is shown in blue.

To further demonstrate the range of the model, we considered 4 other populations to estimate in our example country in sub-Saharan Africa: FSW and MSM in location B and FSW and MSM in location C (Figures 3 and 4). For each population, we consider an unscaled consensus estimate (each estimate’s confidence level is set to *c_j_*=1 as well as a confidence-scaled estimate, where the confidence levels for each estimate are selected by expert opinion. The priors, estimates, and consensus estimates for these 4 populations are shown in Table 2. These 4 estimates show that the effects of confidence scaling are not limited to always increasing or decreasing the consensus estimate. Using confidence scaling over the unscaled version of the model, the point estimate for the population size decreased by 3% for FSW in location B, decreased by 14% for MSM in location B, increased by 13% for FSW in location C, and increased by 1% for MSM in location B. Furthermore, the magnitude of difference in the estimates for FSW in location C and MSM in location B shows that confidence scaling can have a major effect on the estimates.

The unaccounted-for variation for both the confidence-scaled and unscaled estimates for each population also shows the positive effects of having expert-selected confidence levels for the estimates. For the unscaled estimates, the proportion of estimate variation owing to unaccounted-for study bias is 0.89 for FSW in location B, 0.84 for MSM in location B, 0.90 for FSW in location C, and 0.91 for MSM in location C. For the confidence-scaled estimates, the proportion of estimate variability attributable to unaccounted-for study bias is 0.0.11 for FSW in location B, 0.02 for MSM in location B, 0.16 for FSW in location C, and 0.23 for MSM in location C. These results indicate that for the confidence-scaled models, there is less unexplained variation in the *y_j_*s owing to between-estimate variation when compared with the unscaled models.

## Discussion

### Principal Findings

In this study, we presented a Bayesian hierarchical model for triangulating estimates of public health interest, with our examples focusing on PSE. It is a major challenge for stakeholders to come up with a single population size estimate for a given population informed by multiple, potentially contradictory, estimates. Approaches used in the past could be overly simplistic, such as the use of a simple median, or opaque, with stakeholders picking a number after a discussion with no record of their full reasoning.

The model we have proposed assists and formalizes the triangulation process. It makes the decision for a consensus estimate from stakeholders transparent and explicit, while allowing them to retain control over how credible they find each individual estimate. Furthermore, the tool we developed to implement this model has a built-in assessment of the consensus process in the form of unaccounted-for variation; thus, the Shiny app can provide instant feedback on how successfully the individual estimates were combined in consensus. For users working with population size estimates, we recommend rounding results when presenting them to stakeholders to avoid any confusion stemming from noninteger counts of people. For all estimates, we strongly encourage transparency by presenting confidence scores with a rationale for each.

We presented a use case that represents a common challenge: the need to synthesize multiple estimates, often derived from methods of varying quality. In our example, we had the benefit of all empirical PSE and knowing the limitations and errors made during survey implementation. This provided a valuable context for confidence scoring, which affects the consensus estimate. When this information on implementation is not available, the next best option may be to base confidence scores on the strength of the method used to produce each estimate. For example, we presented the PSE derived from events, and all were smaller than the survey sample size. We did not need to know about the implementation errors to know these were nonsensical results that merited very low confidence scores. The PSE were all produced from the same survey; therefore, there were no differences in the age of our PSE to impact confidence scores (eg, we would have low confidence in older PSE and high confidence in the current PSE). As described earlier, our priors were several years old and based on stakeholder consensus. We were not present for the discussions years before our survey and do not know whether this consensus effort was influenced by a few with strong voices or more representative of the entire group of experts. Analysis of our data presented in Figures 3 and 4 suggests that the more disparate our individual estimates were, the more influential the prior was in the consensus estimate. Therefore, we encourage users to gather as much information as possible on the quality of the prior and how it was derived to provide context for the consensus estimate.

### Strengths and Limitations

Other Bayesian approaches such as the *Anchored Multiplier* model [17,18] have been proposed for PSE. Our model differs from the Anchored Multiplier (and its *Variance-Adjusted* counterpart) in 4 ways. First, our model can directly handle any estimated quantity, including both population proportions and absolute sizes from any PSE technique, whereas the Anchored Multiplier is designed for estimates of population proportions and requires the selection of denominators to handle absolute sizes. Second, the Anchored Multiplier uses a binomial distribution as the sampling distribution and a beta distribution as the prior distribution, whereas our model uses normal distributions for both the sampling and prior distributions, with transformations tailoring the model to the estimate type. The effect of this difference in model structure is explored in detail in Multimedia Appendix 1 [18]; the differing structures give the models strengths in different cases, and as such, they represent complementary rather than competing approaches. Third, in the variance-adjusted case, the Anchored Multiplier uses a frequentist estimator of τ^2^ to extend the CIs of the estimates, whereas in our model, τ is a parameter that is fit during the sampling process. This distinction is important, as the DerSimonian-Laird estimator [25] used by the Anchored Multiplier has the potential to underestimate the true value of tau squared [26] and produces confidence (or credible) intervals that are overly narrow, particularly when the number of estimates is small or the true between-study variance is large [27], both of which are frequently encountered when dealing with PSE. A wholly Bayesian approach, such as the one offered by the Triangulator, may better account for between-study variance [27]. Fourth, and most critically, our model provides users with a clear and transparent platform to input their confidence for each estimate, which is displayed alongside the estimates data and consensus results on the final tab of the Shiny app. This is in contrast with the Anchored Multiplier and other techniques, where any adjustments to the uncertainty surrounding the estimates must be made before entering the data, which may lead to confusion among observers regarding the source of the data.

Results from the Triangulator are subject to several user limitations. First, if a user lacks knowledge of the quality of the estimates to be synthesized by the Triangulator, the confidence scores may not reflect the actual quality. High scores given to poor-quality data in the presence of other confidence-scaled estimates may result in a biased consensus estimate, as can low scores given to high-quality data. Next, users may try to adjust the confidence scores and model parameters after running the models to fit a specific, desired consensus estimate. Whether this affects the Triangulator more than similar models is debatable; the Triangulator, the data, confidence scores, and prior information are all transparently presented in the app, whereas with other methods, the inputs may be obfuscated. We provided tutorials for the proper use of the Triangulator to avoid spurious results by making such adjustments.

### Conclusions

As a user-friendly app, the Triangulator has broad utility for statisticians, epidemiologists, and other public health officials—anyone seeking to combine multiple estimates of population size, incidence, prevalence, or other quantities into a single consensus estimate. It offers a solution to the long-standing problem of synthesizing multiple estimates, potentially leading to more informed and evidence-based decision-making processes. Single-point estimates are widely used in decision-making, resource allocation, and policy development. Ministries of health rely on them to meet global health reporting requirements. Humanitarian organizations and multilateral government donors use single-point estimates to establish targets and assess their performance against those targets. The Triangulator leverages the user’s knowledge of the quality of the original estimates as well as prior knowledge of the quantity being estimated. Although technical parameters can be adjusted to meet the needs of certain users, for most use cases, no additional information is required outside of the estimates to be combined (and their uncertainty), confidence scores for each estimate, and parameters to set the prior on the quantity of interest. The functionality increases accessibility to public health teams that may not have statistical support but need to synthesize multiple estimates into a single estimate with uncertainty bounds. The Triangulator has the flexibility to be adapted and used in various other contexts and regions to address similar challenges.

Software, including the Shiny web app source code, is freely available and can be accessed on the internet [28]. The Shiny app is hosted at Epi apps for convenient use [29].

## Appendix

Multimedia Appendix 1 Derivations of the model equations, a sensitivity analysis of the posterior estimate of tau, and a comparison of the model presented in this manuscript to an existing model in the literature.

## Abbreviations

ART: antiretroviral therapy
BBS: biobehavioral survey
FSW: female sex workers
KP: key populations
MSM: men who have sex with men
PSE: population size estimation
SS-PSE: successive sampling population size estimation

## Data Availability

All data generated or analyzed during this study are included in this published article.
