# Supplementary material for: Triangulating Truth and Reaching Consensus on Population Size, Prevalence, and More: Modeling Study
Source: JMIR Public Health Surveill. 2024 Mar 19;10:e48738. doi: 10.2196/48738 (PMC10988376; doi:10.2196/48738)
Supplement: Multimedia Appendix 1 [file publichealth_v10i1e48738_app1.docx]

# Supplementary Material

# Triangulating Truth: A Flexible Statistical Tool for Combining Estimates of Population Size, Prevalence and More

In this supplement, we derive the reduced model, the formula for the data-level unaccounted-for variation of the reduced confidence-scaled model, present brief sensitivity results on the multiplier that sets the scale parameter for the prior on $\tau$, and compare the performance of the Triangulator to that of the Anchored Multiplier.

## Derivation of Reduced Model

The basic model is

$y_{j}$| $\nu_{j},\sigma_{j} \sim N\left( \nu_{j}, \left( \frac{\sigma_{j}}{c_{j}} \right)^{2} \right)$

and

$\nu_{j}$|$\theta,\tau\sim N\left( \theta,\tau^{2} \right)$.

We may write $y_{j}$ as $y_{j}=\nu_{j} + \epsilon_{j}$, where $\epsilon_{j} \sim N\left( 0,\left( \frac{\sigma_{j}}{c_{j}} \right)^{2} \right)$ is independent of $\nu_{j}$. Because $y_{j}$ is the sum of two independent normal distributions it is itself normal, with

$E\left( y_{j} \mid\sigma_{j},\theta,\tau\right)=E\left( \nu_{j} \mid\theta,\tau\right)+E\left( \epsilon_{j} \right)=\theta$

and

$\text{var}\left( \text{y}_{\text{j}} \mid\sigma_{j},\theta,\tau\right)=\text{var}\left( \nu_{j} \mid\theta,\tau\right)+\text{var}\left( \epsilon_{j} \right)=\left( \frac{\sigma_{j}}{c_{j}} \right)^{2}+\tau^{2}$

Therefore

$y_{j}$|$\sigma_{j},\theta,\tau\sim N\left( \theta, \left( \frac{\sigma_{j}}{c_{j}} \right)^{2}+\tau^{2} \right)$.

## Derivation of Unaccounted-for Variation Formula

First, we prove the following lemma:

**Lemma:** Let $x_{1},\ldots, x_{N}$ be $N$ random variables. As well, let $\bar{x}=\frac{1}{N}\sum_{i=1}^{N} x_{i}$ and let $\bigvee_{i=1}^{N} x_{i}=\frac{1}{N-1}\sum_{i=1}^{N} \left( x_{i}^{2}-\bar{x}^{2} \right)$ be the sample variance operator. Then,

$$E\left( \bigvee_{i=1}^{N} x_{i} \right)=\bigvee_{i=1}^{N} E\left( x_{i} \right)+\frac{1}{N}\sum_{i=1}^{N} \text{Var}\left( x_{i} \right).$$

**Proof:** We have

$$E\left( \bigvee_{i=1}^{N} x_{i} \right)=E\left( \frac{1}{N-1}\sum_{i=1}^{N} x_{i}^{2}-\bar{x}^{2} \right)$$

$$=\frac{1}{N-1}\sum_{i=1}^{N} E\left( x_{i}^{2} \right)-E\left( \bar{x}^{2} \right)$$

$$=\frac{1}{N-1}\sum_{i=1}^{N} \text{Var}\left( x_{i} \right)+E\left( x_{i} \right)^{2}-\left( \text{Var}\left( x_{i} \right)+E\left( \bar{x} \right)^{2} \right)$$

$$=\frac{1}{N-1}\sum_{i=1}^{N} \left( E\left( x_{i} \right)^{2}-E\left( \bar{x} \right)^{2} \right)+\frac{1}{N-1}\sum_{i=1}^{N} \left( \text{Var}\left( x_{i} \right)-\text{Var}\left( \bar{x} \right) \right)$$

$$=\bigvee_{i=1}^{N} {E(x}_{i})+\frac{1}{N-1}\left( \sum_{i=1}^{N} \text{Var}\left( x_{i} \right)-N\cdot\text{Var}\left( \bar{x} \right) \right)$$

$$=\bigvee_{i=1}^{N} {E(x}_{i})+\frac{1}{N-1}\left( \sum_{i=1}^{N} \text{Var}\left( x_{i} \right)-\frac{1}{N}\sum_{i=1}^{N} \text{Var}\left( \bar{x} \right) \right)$$

$$=\bigvee_{i=1}^{N} {E(x}_{i})+\frac{1}{N}\sum_{i=1}^{N} \text{Var}\left( x_{i} \right).$$

**▪**

Next, we rewrite the expression for $R^{2}$and apply the well-known fact that for random variables $X$ and $Y$, we have $E\left( X \right)=E\left( E\left( X | Y \right) \right)$. We follow this by applying Lemma 1:

$$R^{2}=1-\frac{E\left( \bigvee_{j=1}^{N} y_{j}-\nu_{j} \right)}{E\left( \bigvee_{j=1}^{N} y_{j} \right)}$$

$$=\frac{E\left( \bigvee_{j=1}^{N} y_{j}-\nu_{j}+\nu_{j} \right)-E\left( \bigvee_{j=1}^{N} y_{j}-\nu_{j} \right)}{E\left( \bigvee_{j=1}^{N} y_{j} \right)}$$

$$=\frac{E\left( \bigvee_{j=1}^{N} \nu_{j} \right)}{E\left( \bigvee_{j=1}^{N} y_{j} \right)}$$

$$=\frac{E\left( E\left( \left. \bigvee_{j=1}^{N} \nu_{j} \right|\theta,\tau\right) \right)}{E\left( \bigvee_{j=1}^{N} y_{j} \right)}$$

$$=\frac{E\left( \bigvee_{j=1}^{N} E\left( \left. \nu_{j} \right|\theta,\tau\right)+\frac{1}{N}\sum_{j=1}^{N} \text{Var}\left( \left. \nu_{j} \right|\theta, \tau\right) \right)}{E\left( \bigvee_{j=1}^{N} y_{j} \right)}$$

where $E(\cdot)$ and $\text{Var}(\cdot)$ are the posterior expectation and variance respectively. Next, we note that in the full confidence-scaled model, the posterior distribution for $\nu_{i}$ is given by

$$\nu_{i}|\theta,\tau\sim N(\hat{\nu}_{i},V_{i})$$

Where $\hat{\nu}_{i}=\frac{\tau^{2}}{\tau^{2}+\sigma_{i}^{2}}y_{i}+\frac{\sigma_{i}^{2}}{\tau^{2}+\sigma_{i}^{2}}\theta$ and $V_{i}=\frac{\tau^{2}\sigma_{i}^{2}}{\tau^{2}+\sigma_{i}^{2}}$. Thus, we have

$$R^{2}=\frac{E\left( \bigvee_{j=1}^{N} \hat{\nu}_{j}+\frac{1}{N}\sum_{j=1}^{N} V_{j}\text{ } \right)}{E\left( \bigvee_{j=1}^{N} y_{j} \right)}.$$

## Sensitivity of Posterior Estimate of $\tau$ to “$\tau$ multiplier”

Included below are plots of the median and 95% CI for the posterior distribution of $\tau$, as well as $R^{2}$, as the $\tau$-multiplier (which sets the scale parameter on the prior for $\tau$) varied from 0.0001 to 1000 for FSW estimates in Locations A, B, and C. As one can see, our choice of default multiplier (0.1) offers a compromise between highly-restrictive $\tau$ priors (e.g. a multiplier of 0.0001) and non-informative $\tau$ priors (e.g. a multiplier of 1000)

| 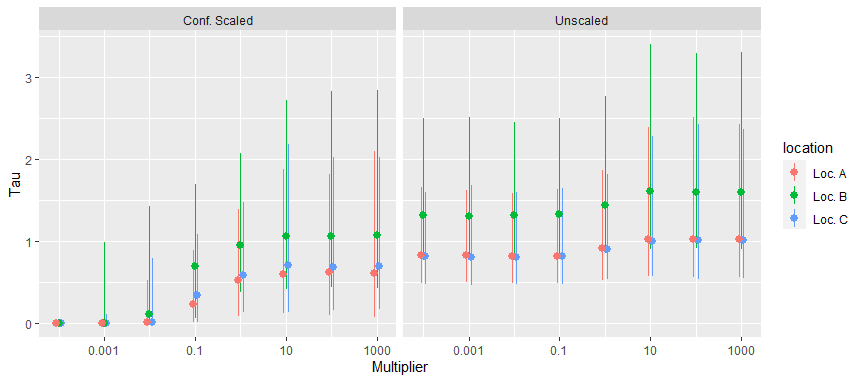 |
| --- |
| (a) |
| 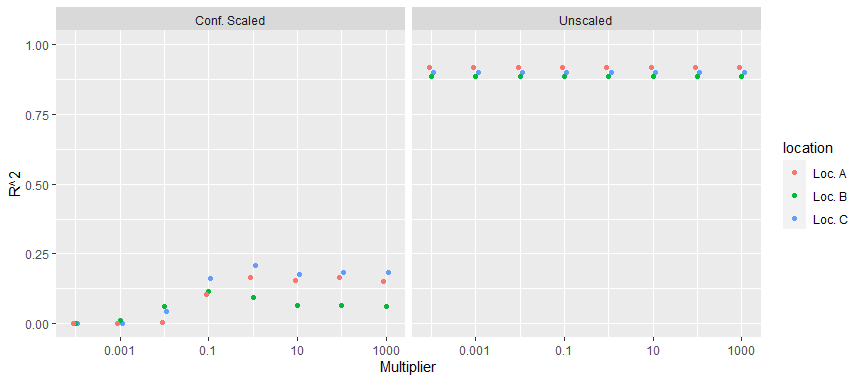 |
| (b) |
| **Figure 1**. Plots of (a) the median and 95% CI for the posterior distribution of $\tau$and (b) $R^{2}$ as the $\tau$ multiplier (which sets the scale parameter on the prior for $\tau$) varied from 0.0001 to 1000 for FSW estimates in Locations A, B, and C. Note that $\tau$ was estimated for log-transformed data. |

## Comparison to the Anchored Multiplier

As the Triangulator is similar to the Anchored Multiplier in both purpose and design, we compare their performance on selected result from the main text and point out a situation where their results may diverge.

First, we provide a sketch of an explanation as to what situations might lead to differing results between the two methods. Let $\theta$ be the true value quantity of interest, such as population size. Let $\mu_{0},\sigma_{0}^{2}$ be the prior mean and standard deviation of $\theta.$ Let $\mu_{i}$ be the observations of the quantity of interest (with $i=1,\ldots,n)$, each with a standard error of $\sigma_{i}$. The model presented in the main paper uses a normal prior distribution and a normal sampling distribution:

$$\mu_{i}\sim N\left( \theta,\sigma_{i}^{2} \right)$$

$$\theta\sim N(\mu_{0},\sigma_{0}^{2})$$

A well-known result is that posterior distribution of $\theta$ will also be normal (as the sampling and prior distributions are conjugate) with

$$\theta|\mu\sim N\left( V^{-1}\cdot\sum_{i=0}^{n} \frac{\mu_{i}}{\sigma_{i}^{2}},V^{-1} \right)$$

where $V=\sum_{i=0}^{n} \sigma_{i}^{-2}$. Thus, we can write the posterior expectation of $\theta$ as

$$E\left[ \theta| \mu\right]=\sum_{i=0}^{n} v_{i}\mu_{i}$$

where $v_{i}=\frac{\sigma_{i}^{-2}}{\sum\sigma_{i}^{-2}}.$ Thus, the posterior estimate for $\theta$ can be viewed as a weighted average of the $\mu_{i}$ with weights $v_{i}$.

The Anchored Multiplier model [1] uses a binomial sampling distribution and beta prior distribution of $\theta:$

$$\alpha_{i} \sim\text{Binom}\left( n_{i},\theta\right)$$

$$\theta\sim\text{Beta}(\alpha_{0},\beta_{0})$$

where $\alpha_{i}= -\frac{\mu_{i}\left( \sigma_{i}^{2}+\mu_{i}^{2}-\mu_{i} \right)}{\sigma_{i}^{2}}, \beta_{i}= \frac{(\mu_{i}-1)\left( \sigma_{i}^{2}+\mu_{i}^{2}-\mu_{i} \right)}{\sigma_{i}^{2}}$, and $n_{i}=\alpha_{i}+\beta_{i}=\frac{\mu_{i}\left( 1-\mu_{i} \right)-\sigma_{i}^{2}}{\sigma_{i}^{2}}$. Furthermore, it follows that $\alpha_{i}=\mu_{i}n_{i}$ and $\beta_{i}=\left( 1-\mu_{i} \right)n_{i}$. As the beta distribution is the conjugate prior of the binomial distribution, the posterior will also be a beta distribution with the following well-known form:

$$\theta|\mu\sim\text{Beta(}A,B\text{)}$$

where $A=\sum_{i=0}^{n} \alpha_{i}$ and $B=\beta_{0}+\sum_{i=1}^{n} n_{i}-\alpha_{i}=\sum_{i=0}^{n} \beta_{i}$. Thus, the posterior expectation of $\theta$ is

$$E\left[ \theta| \mu\right]=\frac{A}{A+B}=\frac{\sum_{i=0}^{n} {n_{i}\mu}_{i}}{\sum_{i=0}^{n} {n_{i}\mu}_{i}}=\sum_{i=0}^{n} {w_{i}\mu}_{i}$$

where $w_{i}=\frac{n_{i}}{\sum n_{i}}$. Thus, the posterior estimate of $\theta$ can also be viewed as a weighted average, but with weights $w_{i}$. From this, we see that in computing the posterior estimate of $\theta$, one situation where the Anchored Multiplier weights observations more heavily is when the observation is near 0.5 and the standard error is low.

To illustrate the effect, we consider the case of Location B FSW again (Figure 2). Here, we do not transform the data for the Triangulator, and convert all estimates to proportions for the Anchored Multiplier, and then convert back after combining them. Applying both methods, as well as the variance-adjusted anchored multiplier, both with and without confidence-scaling, we see that the results of all methods are relatively consistent with one another. However, when an extreme example is added in (1/3 the size of the reference population of about 70,000 – 25% confidence score), the Anchored Multiplier result is affected significantly. Both confidence scaling and variance-adjustment do have a mitigating affect, but a takeaway is clear: that the models and their adjustments complement each other, and have different strengths.

| 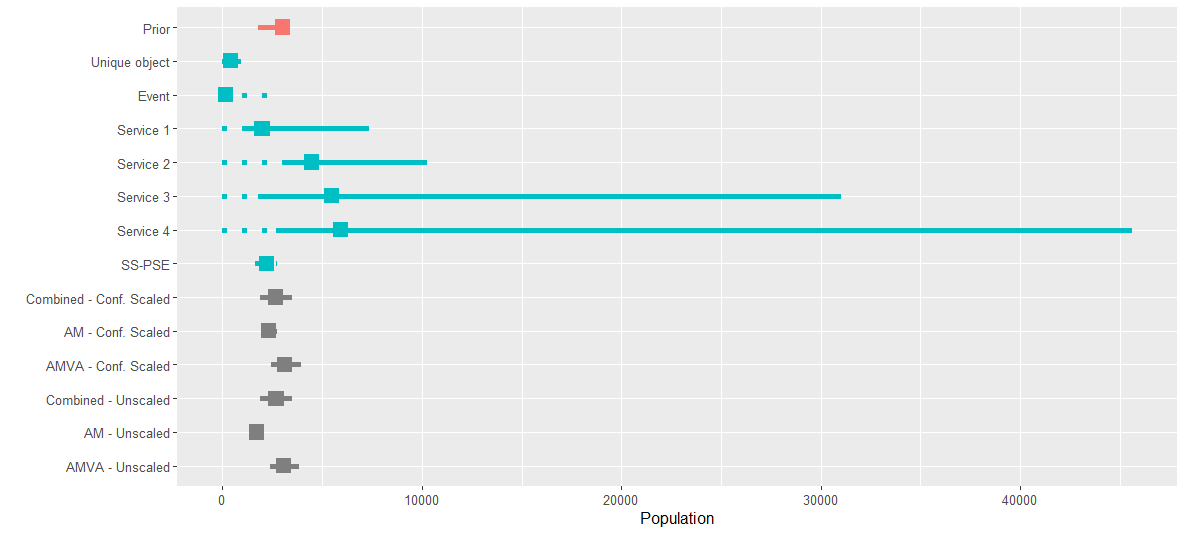 |
| --- |
| (a) |
| 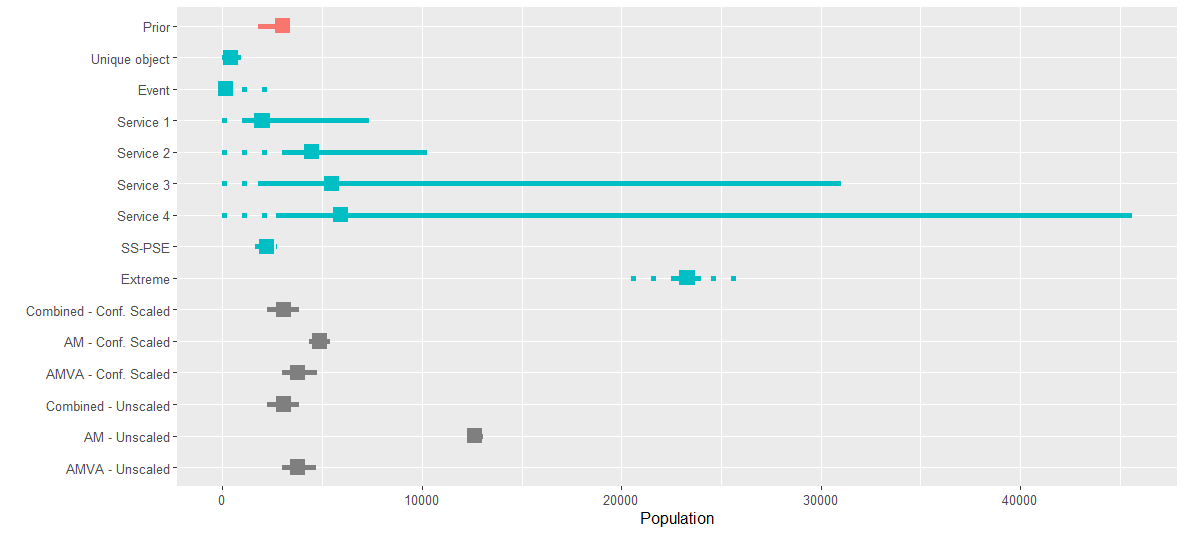 |
| (b) |
| **Figure 2**: Comparison of the results of the Triangulator (“Combined”) and Anchored Multiplier, both unadjusted (“AM”) and variance-adjusted (“AMVA”), both with and without confidence scaling, for Loc. B FSW (a) and for Loc. B FSW with a fictional, extreme observation added (b). The extreme example was chosen to have a mean of 1/3 of the reference population, standard error of 1/100 the reference population, and a confidence score of 25%. No transformation was applied when using the Triangulator, while the results were converted to proportions for the Anchored Multiplier, and then converted back after the estimates were combined. The size of the reference population was approximately 70,000. |

1. Wesson PD, Mirzazadeh A, McFarland W. A Bayesian approach to synthesize estimates of the size of hidden populations: the Anchored Multiplier. Int J Epidemiol. 2018;47(5):1636-44.
